# Pattern of medication selling and self-medication practices: A study from Punjab, Pakistan

**DOI:** 10.1371/journal.pone.0194240

**Published:** 2018-03-22

**Authors:** Muhammad Majid Aziz, Imran Masood, Mahreen Yousaf, Hammad Saleem, Dan Ye, Yu Fang

**Affiliations:** 1 Department of Pharmacy Administration and Clinical Pharmacy, School of Pharmacy, Xi’anJiaotong University, Xi’an, China; 2 Center for Drug Safety and Policy Research, Xi’an Jiaotong University, Xi’an, China; 3 The Global Health Institute, Xi’an Jiaotong University, Xi’an, China; 4 Shaanxi Center for Health Reform and Development Research, Xi’an, China; 5 Faculty of Pharmacy and Alternative Medicine, The Islamia University of Bahawalpur, Bahawalpur, Punjab, Pakistan; 6 Faculty of Pharmacy, Bahauddin Zakariya University, Multan, Pakistan; 7 Institute of Pharmaceutical Sciences, University of Veterinary & Animal Sciences, Lahore, Pakistan; Fundació Institut Català de Farmacologia, SPAIN

## Abstract

**Background:**

Access to medicines without prescription is a major contributing factor for self-medication practices. This study was designed to examine the ratio of non-prescribed medicines sales and self-medication practices in Punjab, Pakistan. This study also evaluates the reasons for self-medication within its communities.

**Methods:**

An observational study was conducted in 272 systemically selected pharmacies to analyze medicines-related sales, with or without prescription. A cross-sectional survey was performed between June 2015 and November 2016. Consumers were interviewed about their self-medication practices.

**Results:**

Of the pharmacies surveyed, 65.3% participated in the study. A total of 4348 medicines were purchased for self-medication by 3037 consumers (15.2% of all study participants), of which 873 (28.7%) participated in an interview. Majority (81.2%) medicine purchaser, (90.9%) interview participants, and (59.4%) drug users were male. On average, each community pharmacy sold 7.9 medicines without prescription each day, to an average of 5.5 customers. Many participants (28.9%) had matriculation in their formal education. The medicines most often sold for self-medication were analgesics and antipyretics(39.4%). More than 25% of participants reported fever symptoms and 47.8% assumed their illness was too trivial to consult a doctor. Media advertisements were the most common source of information for participants (46.7%).

**Conclusion:**

Many types of medicines were often sold without prescription from community pharmacies. Self-medication was common practice for a wide range of illnesses. Pakistan also needs effective implementation of policies to monitor medication sales. Public education about rational medication and limits to advertising medicine are very necessary.

## Introduction

Self-medication is a common practice in healthcare system. People want to self-care using medicines following self-diagnosis. Management of minor ailments can be achieved using medicinal and non-medicinal agents. It also relates to the use of first-aid in everyday life [1]. Socioeconomic culture, personal traits, and healthcare system play an important role in self-medication prevalence and practice [2]. Self-medication has some pros and cons. It rivets some risks to human: consumers may incur significant financial costs; excessive antimicrobial use can lead to pathogen-resistance; many adverse drug events and drug–drug interactions can occur; and life expectancy can shorten [3]. However; appropriate self-medication can save time and money, empower public to cure minor ailments themselves, and increase public confidence for making decisions to improve their own health [4]. Self-medication is global practice regardless of prescription cost and outline of health care system [2,3,5–17]. Self-medication is general practice in developed nations such as Australia, Italy and Spain [2, 5, 6]. Similarly, people in middle-income countries also prefer to self-medicate, for example in Brazil and China [7, 8]. Not surprisingly, health issues are often managed through self-medication in Egypt, Ethiopia, India, Indonesia, Kenya, Mongolia, Nepal, Tanzania, Serbia and Zimbabwe—all low to middle-income nations [3, 9–17]—Pakistan is no exception in this regard.

In Pakistan, about 79% primary care is provided by private sector that can be a cause of self-medication practices [18]. The contributing factors to the high prevalence of self-medication in Pakistan include: ease of access to medicines, lack of healthcare knowledge, excessive marketing, inadequate enforcement of regulatory policies, poor accessibility to healthcare providers, and lack of public healthcare facilities [19–22]. Access to medication is a key contributing factor of high prevalence of self-medication practices [23]. More than half of all medicines sold in Pakistan are supplied without prescription [24]. Analgesics, antibiotics, anti-diarrheal agents, antihistamines, antipyretics, cough-suppressants, ‘tonics’, and vitamins are readily available without prescription from community pharmacies in Pakistan [19–22]. Due to elevating sale of non-prescribed medicines self-medication rate is steadily increasing in Pakistan [21–23]. Still; there is no national and provincial study. We examined pattern of medication selling in community pharmacies and evaluated self-medication practices in Punjab, Pakistan.

## Materials and methods

### Study setting

Study sites were community pharmacies in Punjab, Pakistan. The area of the Punjab province is 205,344 square kilometers and is the most populous province. The population is estimated to be more than 91 million, or 56% of the total national population [25].

### Study design, sampling and analysis

A two-phase cross-sectional study was performed between June 2015 and November 2016. Moreover; the protocols, pattern, and sample size was initially modified according to the result of a pilot study.

#### Phase 1

Licensed community pharmacies (n = 272) were systemically selected (S1 File) from a list of all pharmacies within the province. To ensure the equal number of pharmacies according to population and total number of pharmacies from each community setting^__^ rural to big cities: quota sampling technique was used. Each pharmacy was given an identification number, then observed to determine the total number of consumers presenting with or without prescription/s. Due to working feasibility of pharmacies, each pharmacy was observed for 2 days. The total number and type of medicines that were sold without prescription were calculated by using data collection form (S2 File). Finally, patterns were recorded about how consumers asked to buy medicines for self-medication. The contact and address of consenting consumers of each city were compiled separately. The phone numbers of agreed consumers were also collected to know their feasibility of location and time of interview.

#### Data collectors

Two trained data collectors were appointed at each sale counter in pharmacy. One data collector was assigned to assess the sale pattern and recruitment of customers for further interviews. The second data collector was responsible to analyze the purchaser's pattern of request to acquire medicine without prescription. The reliability of each data collector was assessed using "percent agreement" by trainer in a pilot study.

#### Phase 2

Purchasers of medicines for self-medication were invited for face to face interview. Consumers were interviewed about their reasons for self-medication, and the information sources they had used. During the phase 1, it was found that minimum four customers from some pharmacies were willing for interview. To ensure the equal participation from each pharmacy, four consumers were targeted from each selected pharmacy. The sample size of 1088 consumers was estimated on the basis of minimum consenting consumers from a pharmacy during entire study. But on contact, many consenting consumers were unavailable. The inclusion criteria were revised that ensure the minimum two customers from each pharmacy.

#### Questionnaire

A semi-structured questionnaire (S3 File) was developed from previous studies [2,7–16,19–22]. The initial questionnaire was developed in English then translated into Urdu (national language). The accuracy of translation was verified by three language experts.

Statistical Package for Social Sciences (SPSS) version 18.0 was used for descriptive analyses.

#### Pilot study

The accuracy and consistency of data collection form and reliability of data collectors was assessed in a pilot study of 20 pharmacies. Each pharmacy was observed for one day. The response of pharmacies to participate in study was also estimated to calculate the sample size. The standardized questionnaire was tested in 30 participants before actual data collection and modified on basis of received feedback.

### Ethical consideration

The study’s design and protocols were approved by the Center for Drug Safety and Policy Research at Xi’an Jiaotong University after the permission of Ethics Review Committee at Xi’an Jiaotong University, China (Ref # MR102-15/Phar) and Pharmacy Research Ethics Committee at The Islamia university Bahawalpur, Pakistan (Ref # 67-2015/PREC); however, before data collection a written or verbal consent was taken from consumers (S4 File) and the pharmacy retailers/pharmacists (S5 File). All study participants were fully aware and informed about the study’s purpose. The data contained non-identifiable details with the names of participants and pharmacies anonymized. Identification numbers were used to conduct the interviews, collect data, and monitor progress.

## Results

Total 417 pharmacies were contacted to get sample of 272 pharmacies and 145 (34.8%) pharmacies refused to participate in the study. About 44.6% (29) pharmacies from suburban and rural areas refused to participate. Thirty-seven (40.6%) pharmacies from tehsil cities, 43 (37.4%)pharmacies from district cities, and 36 (24.7%)pharmacies from divisional cities refused their participation.

### Phase 1: Sale of non-prescribed medicines

During the study, 4886 of 19877 customers (24.5%) presented without a prescription. Of this cohort, 3037 (62.2%) of customers purchased without any advice, 1236 (25.3%) received advice from an unregistered medical practitioner, and 613 (12.5%) were regular customers of the relevant pharmacy.

Further analysis shows that 3037 of 19877 customers (15.2%) had purchased medicines for self-medication. Among these, 573 (18.8%) were female. An average of 5.5 customers per day visited each pharmacy to purchase medicines for self-medication. Many customers (41.9%) purchased medicines by mentioning the name of medicine as given in Table 1.

**Table 1:** Type of requests to acquire medicines (n = 3037).

Modes of request	Frequency	Percentage
Mentioned the name of medicine	1275	41.9%
Mentioned the name of drug category	964	31.7%
Showed the prior used medicine or their package	582	19.1%
Mentioned the physical characteristics of medicine	216	7.1%

**Fig 1:**
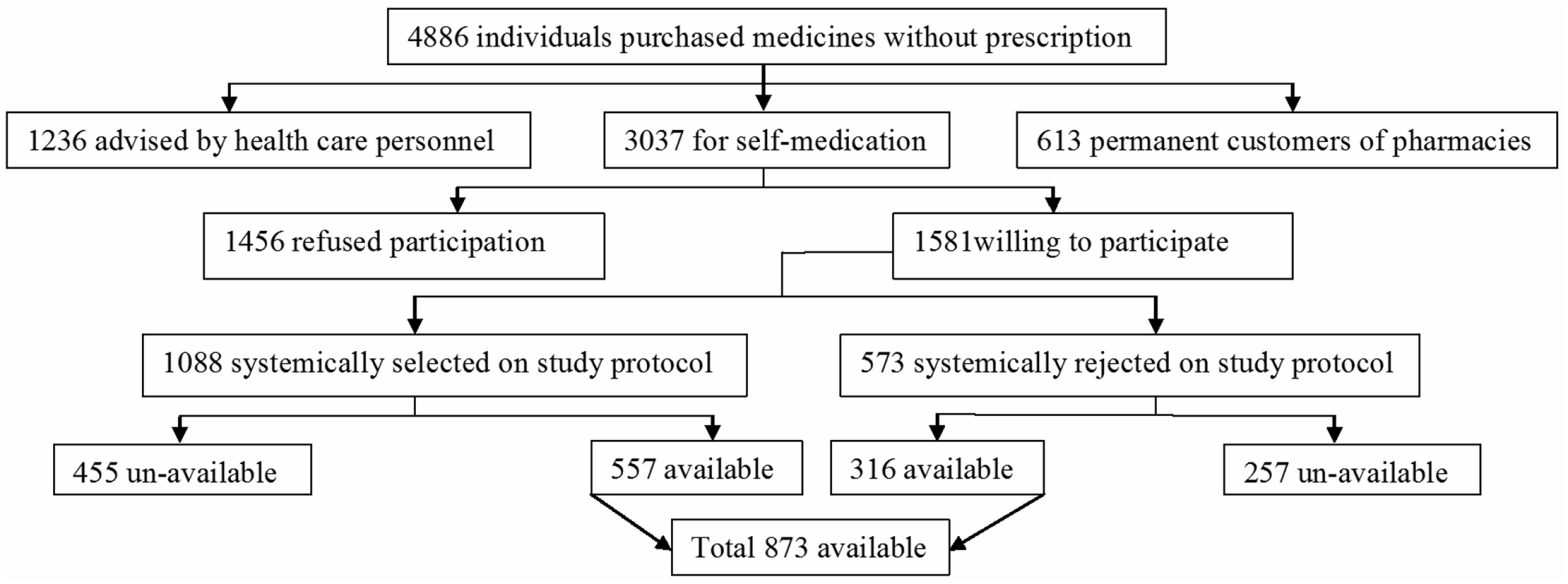
An outline of study.

Of the 3037 customers, 1581 (52.0%) were willing to be interviewed at the time of visit. According to the study protocol, 1008 customers (63.8%) were selected, but only 557 (55.2%) were available for interview. Thus; we included all willing purchasers. In summary, 873 of 3037 customers (28.7%) were available for interview at contact (Fig 1).

**Fig 2:**
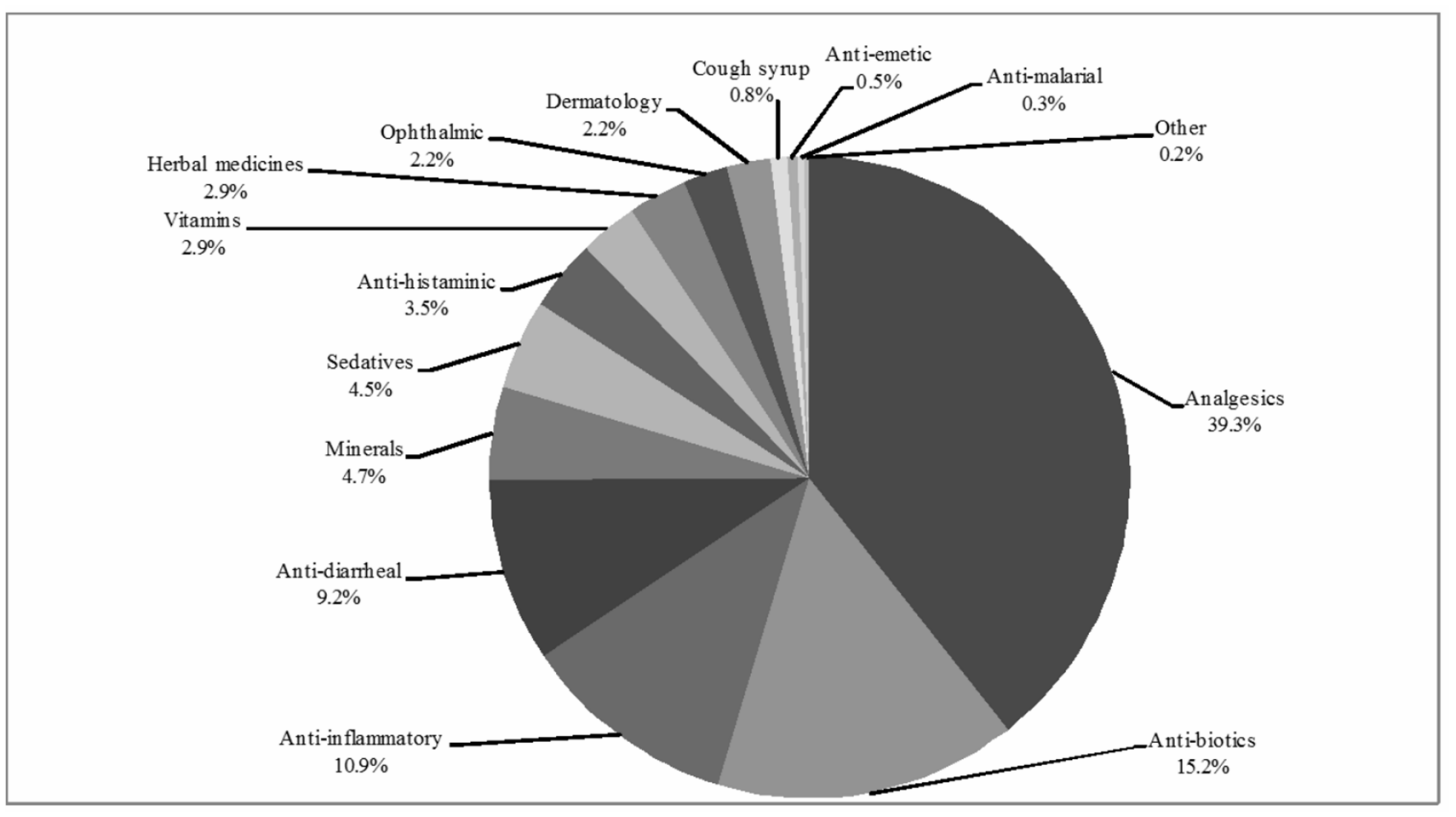
Sale of non-prescribed medicines.

A total of 4348 medicines were purchased for self-medication, including OTC and prescription-only-medicines (POM), Fig 2. Averages of 7.9 medicines per day were sold by each pharmacy without prescription: 1712 analgesics and antipyretics (39.4% of total), 662 antibiotics (15.2%), 478 anti-inflammatory agents (10.9%), and 403 anti-diarrheal medicines (9.3%) were purchased.

### Phase 2: Self-medication practices

Of 873 medicine users, 19 (2.2%) were pregnant women, 25 (2.8%) were breast-feeding, 23 (2.6%) were diabetic, 38 (4.3%) were hypertensive, and 6 (0.7%) were old with poor health. Among pregnant women, about 10.5% used an herbal product named as Joshanda (S6 File) and 5.3% used metronidazole. The use of metoclopramide (4.3%) and aspirin (4.3%) was also observed in breast-feeding mothers. Similarly, 7.9% hypertensive costumers purchased ibuprofen.

Further, in combination with allopathic medicines, 37 consumers (4.2%) were self-medicating with herbal medicines, and 15 (1.7%) were taking homeopathic medicines. A total of 208 (23.9%) customers revealed during the interview that they had purchased the medicines for use in children.

#### Socio-demographic characteristics

The mean age of participants was 35.0±2.8 years; 27.0% of the total study group was aged 31–35 years; and 90.9% were male. Up to 28.9% of participants had matriculation in their formal education; 49.9% had a monthly income of PkR 15001–30000 (approximately USD 145–290); and 46.7% were living with 5–7 family members (Table 2).

**Table 2:** Socio-demographic characteristics of participants (n = 873).

Characteristics	Range/ Groups	Frequency	Percentage
Age (years)	25 or below	89	10.2
	26–30	118	13.5
	31–35	236	27.0
	36–40	163	18.7
	41–45	117	13.4
	46–50	92	10.5
	51 or above	58	6.6
Mean age ± SD		35.0±2.8	
Educational status	Below matriculation	154	17.6
	Matriculation	253	28.9
	Intermediate	143	16.4
	Bachelor	194	22.2
	Master	120	13.7
	Higher education	9	1.0
Gender	Male	794	90.9
	Female	79	9.1
Monthly income in Pakistani rupees(PkR)	Less than or 15000	188	21.5
	15001–30000	436	49.9
	30001–45000	149	17.0
	45001–60000	78	8.9
	More than 60000	22	2.5
Family size (Members)	1–4	163	18.7
	5–7	408	46.7
	More than 7	302	34.6

**Fig 3:**
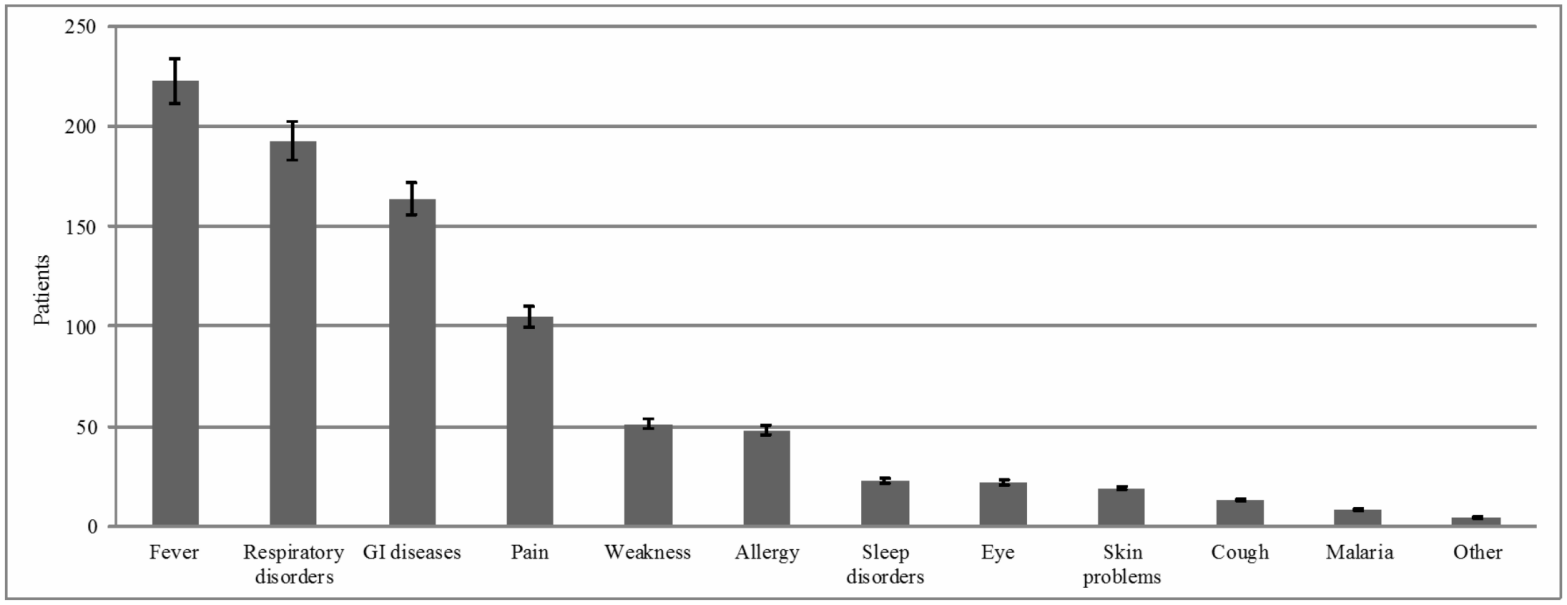
Perception of participants about their disease.

About 59.4% (519) of drug consumers were male and 40.6% (354) were female. Participant perceptions about the disease/s they were trying to ameliorate by self-medicating are described in Fig 3: 223 (25.5%) reported headache or fever, 193 (22.1%) respiratory problems, and 164 (18.7%) gastrointestinal diseases.

The main drivers for self-medicating are listed in Table 3—among them, 417 (47.8%) believed that the illness treated did not warrant a formal consultation with a doctor but 13.7% (57) among them purchased anti-bacterial, anti-malarial, anti-psychotic, ophthalmic, and skin medicines.

**Table 3:** Reason of self-medication (n = 873).

Reason for self-medication	Frequency	Percentage
Insignificant illness	417	47.8
Affordability	185	21.2
Access to hospital (24/7)	112	12.8
To save time	71	8.1
Lack of confidence at doctor	63	7.2
Emergency use	23	2.6
Privacy	2	0.2

Participants used four main sources of information including: media advertisement (46.7%), information from relatives, neighbors and/or friends (21.6%), and the internet (0.3%); 31.3% reported they had self-medicated based on previous experiences.

## Discussion

Access to medicines without prescription sanctions self-medication practice that is a global challenge [6–22]. Like Karachi, many community pharmacies in Punjab sale the medication without prescriptions [26]. The drug sale without prescription and prevalence of self-medication varies from region to region, with its rate of use being driven by societal norms, personal traits, pharmacy staff, and the regulatory policies of the country [3,4,10,13]. This study showed that 15.2% of customers were supplied medication from a pharmacy without a prescription, similar to private pharmacies in Spain and Zimbabwe [6,17]. We observed that more than seven medicines per day were sold without any prescription by each community pharmacy. As a consequence, consumers in Pakistan have free access to all type of medicines. Consumers purchased a variety of POMs for self-medication, treating them like standard OTC medicines. [19–22]. In addition, 34.7% pharmacies refused to participate in this study. This indicates that more POM may sold without prescription by them because pharmacies in Pakistan have only business-oriented approaches [23,26]. The trend of refusal increased from big cities to rural pharmacies as compliance to legal requirement is lower in rural pharmacies of Pakistan [27].

The current study reflects findings seen in other reports from Ethiopia, India and Italy relating to high self-medication rates with analgesics and antipyretics [2,12,13]; these agents are commonly used to treat and manage uncomplicated illnesses such as fever, pain and headache. Globally, analgesics are mostly sold as OTC medicines for self-medication [21,28]. This study demonstrates that analgesics are most preferred medicine for self-medication. Several analgesics combination are available in Pakistan and are frequently used [22,29]. However, conscious use of analgesic combination for pain management is necessary [30]. Simultaneous use of many combinations or overdosing of any combination may cause side effects or cannot provide analgesic effects [31]. This study also shows the insecure use of analgesics in hypertensive patients [32]. The next most popular class of medicines sold for self-medication was non-prescribed antibiotics. The alarming sales of non-prescribed antibiotics account for 15.2%. Antibiotics in the Punjab community are widely misused for ‘colds’, cough, diarrhea, flu, fever, and sore throat [19,33]. An excessive rate of antibiotic self-medication (62%) has been reported in university students of this province [34]. Our study shows similar antibiotic self-medication practices compared with countries such as India, Indonesia, Mongolia and Spain [6,9,11,12]. Self-medication with antibiotics is a considerable health concern because antibiotic-resistance rates are escalating globally [6,9,13,19,22,35].

This study shows that many people in the community rely on herbal and alternative medicines. These were used either alone or in combination with allopathic medicines for self-medication [33]. However, many adverse drug reactions are related to the use of herbal products with modern medication [36]. Approximately 22% of Pakistan’s population use a herbal product known as "Joshanda" (S6 File) [33,37]. In this study, the use of joshanda is also found in10.5% pregnant women. However; some ingredients of joshandalike *Ephedra sinica*, *Foeniculum vulgare*, *Glycyrrhiza glabra*, *Hyssopus officinalis*, and *Mentha piperita* are contraindicated or consider unsafe during the pregnancy [38]. The use of herbs to treat different diseases has been reported in Egypt and China [8,14]. However, herbal and alternative medicines are widely known to have serious safety, quality and efficacy issues [39].

The patterns of medicine sales for self-care reported in this study are similar to those reported in already published international research, especially with regard to non-prescribed anti-diarrheal, antihistaminic, anti-inflammatory, anti-emetic, anti-malarial, anti-mycotics, sedatives and hypnotics, minerals, ophthalmic medicines, skin products, and vitamins. The results of this study reinforce the findings of several previous studies conducted in developing countries that highlight the very frequent use of these medicines without prior advice from a doctor [10,12,13,15,16].

Similar to people in Australia and India, the public of Pakistan also routinely purchase cough syrups for self-care [5,12]. Study participants also frequently purchased non-prescribed medicines for children; this is similar to rates observed in Kenya [10]. Pregnant women in Pakistan were observed to use non-prescribed medicines in a similar fashion to women in India [12]. This study authenticates the use of anti-diarrheal in pregnant women. In addition, the use of anti-emetic and analgesics in breast-feeding mothers is also determined by this study. However; the use of anti-diarrheal agents during pregnancy [40] and anti-emetic and analgesics in breast-feeding mother appends some risks [41,42]. Self-medication practice was seen in consumers with chronic diseases such as diabetes and hypertension. Such practices have numerous risks and can be a health hazard for many consumers because they can adversely affect regular and ongoing therapies being used to treat chronic disease [39,43]. We noted in our study that a major source of self-medication information came from media advertisements. Similar to some other Asian and European countries, medicine companies in Pakistan frequently advertise herbal medicines, analgesics, cough syrups, and antipyretics [12,44,45]. In Khyber-Pukhtoonkhwa, 21% of consumers reported that they had been inspired to self-medicate having seen an advertisement [46].

Access to hospital (24/7) is a powerful trigger for self-medication practices [19–22,46]. Only 45% Pakistani has access to doctors and adequate healthcare facilities. Moreover, less than 21% of population in Pakistan has access to public sector's facilities for primary care [23]. Provision of appropriate health facilities can significantly constrain self-medication practices. Lack of confidence at physician is another cause of self-medication in communities [47]. More than 70% participants of this study have low monthly income. Therefore, affordability also prompts self-medication [18–23]. This study also demonstrates a serious health literacy issue in Pakistan and highlights poor health behaviors concerning medicines management. In contrast to choice of medicines for self-medication, most people assume their illnesses are trivial and would prefer to self-medicate rather than visit a doctor [3,13,22,46]. Many people ignore health issues because of poor knowledge and low literacy [40,48]. Only 1% this study participants have higher education. The low participation of public in this study is due to low education level [49]. Self-medication is increasing because a large proportion of Pakistan society has received very little information about the risks of self-medication [19, 20,35]. The community pharmacies are ideally placed to educate the public about the careful use of medicines [13,45,48]. Community pharmacists in Pakistan must play an effective role in combating excessive self-medication to improve health outcomes and reduce harm [13,22,35]. Further, authorities should implement and closely monitor National Drug Policy (NDP) of Pakistan regarding the sale of OTC and POM from distribution points [50].

## Limitations

This study was conducted in selected pharmacies and willing consumers, which may have introduced bias. Likewise, the many pharmacies refused to participate in the study. The drug selling without prescription may increase in these pharmacies. The self-medication patterns observed in this study related to medicines sales only. The true picture may be different, as many people in Pakistan probably use leftover medicines in their homes.

## Conclusions

This study indicates the sale of many types of medicines without prescription from community pharmacies and signifies poor implementation of NDP. Self-medication practices are common in people across a range of socio-demographic characteristics, demonstrating serious issues of health literacy, affordability and access to health facilities in Pakistan. Broad education programs like health seminars and campaigns in communities should start immediately by government and continue on a regular basis. Government should facilitate public with adequate healthcare services. Pakistan also needs policy execution to monitor medication sales, and to control and regulate the excessive sales of non-prescribed medicines. The advertising of medicines to the public should have limitations.

## Supporting information

S1 File Selection of pharmacy. (PDF)

S2 File Data collection form: Medicines purchased for self-medication from pharmacy. (PDF)

S3 File Interview guide. (PDF)

S4 File Permission and information Sheet/Patients. (PDF)

S5 File Permission and information Sheet/Pharmacy. (PDF)

S6 File Composition of Joshanda. (PDF)
