# Supplementary material for: Pattern of medication selling and self-medication practices: A study from Punjab, Pakistan
Source: PLoS One. 2018 Mar 22;13(3):e0194240. doi: 10.1371/journal.pone.0194240 (PMC5863987; doi:10.1371/journal.pone.0194240)
Supplement: S1 File — (PDF) [file pone.0194240.s001.pdf]

Selection of Pharmacy

1. A list of pharmacies was obtained from department of health or medicine supply companies.
2. Pharmacies are selected from the list by numeric selection (Like every 5th , 8th or 10th depending upon the number of pharmacies in a city)
3. If any pharmacy was not interested to participate in study or had any doubt about the study was not included and another pharmacy with in the area of 2 Km was selected.
4. Pharmacies were selected from the cities of 36 districts and 9 administrative divisions on the following basis by considering administrative division as single unit.
   a. **Divisional city**: 10 pharmacies from divisional head quarter and listed as district A.
   b. **District city** : 8 pharmacies from another district B
   c. **Tehsil City** : 6 pharmacies from other district C.
   d. **Towns and rural areas**: 4 pharmacies selected either from district D.(If any division have not district D then pharmacies in the suburban and rural areas of district A were included)
5. We ensured a homogeneous and uniform presentation of pharmacies from all areas of Punjab.

**Administrative Division (Name)**	**Pharmacies(n) divisional city**	**Pharmacies(n) district city**	**Pharmacies(n) Tehsil city**	**Pharmacies(n) Towns and Rural areas**	**Total Pharmacies**
Bahawalpur	Bahawalpur(10)	Bahawlnagar (8)	Sadiq abad (6)	Yazman (2), Ahmad Pur (2)	28
Dera Ghazi Khan	Dera Ghazi Khan (10)	Muzaffargarh (8)	JamPur (6)	Karor Lal Esan (2) ,Layyah (2)	28
Faisalabad	Faisalabad (10)	Jhang (8)	Chiniot (6)	TT Singh (2), Gojra (2)	28
Gujranwala	Gujranwala (10)	Hafizabad (8)	Daska (6)	Malakwal (2), Shakargarh (2)	28
Lahore	Lahore (30)*	Sheikhupura (8)	Kasur (6)	Chunian (2),Pattoki (2)	48
Multan	Multan (10)	Khanewal (8)	Kahror Pakka(6)	Burewala (2),Mailsi (2)	28
Rawalpindi	Rawalpindi (10)	Attock (8)	Chakwal (6)	Dina (2), Jehlum (2)	28
Sahiwal	Sahiwal (10)	Okara (8)	Chichawatni (6)	Pakpattan (2),Arifwala (2)	28

Sargodha	Sargodha(10)	Bhakkar (8)	Khushab (6)	Piplan (2),Isakhel (2)	28
Grand Total of selected Pharmacies in Punjab province					272

*30 pharmacies are selected from Lahore city because of number of pharmacies and population of city. Thus 3 pharmacies were selected from each town to make sure the equal representation.
