# Supplementary material for: Pattern of medication selling and self-medication practices: A study from Punjab, Pakistan
Source: PLoS One. 2018 Mar 22;13(3):e0194240. doi: 10.1371/journal.pone.0194240 (PMC5863987; doi:10.1371/journal.pone.0194240)
Supplement: S2 File — (PDF) [file pone.0194240.s002.pdf]

Data Collection Form: Medicines Purchased for Self-Medication from Pharmacy

Pharmacy ID #------------------------------- Observer: --------------------------------------- Date: ----------------------------

Patient #	Gender	Name of Medicine 1	Name of Medicine 2	Name of Medicine 3	Name of Medicine 4	If agreed for interview please mention details
P 1						
P 2						
P 3						
P 4						
P 5						
P 6						
P 7						
P 8						
P 9						
P 10						

How patient demands medicines? : Please tick (✓) the relevant

**Way to acquire medicine**	**P 1**	**P2**	**P3**	**P4**	**P5**	**P6**	**P7**	**P8**	**P9**	**P 10**
By mentioning the name of medicine										
By mentioning the name of drug category										
By showing the prior used medicine or their package										
By physical characteristics of medicine										

Number of patients: Hourly record (Please mention the total number of medicine of each hour)

**Hours**	**8.00**	**9.00**	**10.00**	**11.00**	**12.00**	**13.00**	**14.00**	**15.00**	**16.00**	**17.00**	**18.00**	**19.00**	**Total**
With Prescriptions													
Without Prescriptions													
**Hours**	**20.00**	**21.00**	**22.00**	**23.00**	**24.00**	**1.00**	**2.00**	**3.00**	**4.00**	**5.00**	**6.00**	**7.00**	**Total**
With Prescriptions													
Without Prescriptions													
