# Supplementary material for: Pattern of medication selling and self-medication practices: A study from Punjab, Pakistan
Source: PLoS One. 2018 Mar 22;13(3):e0194240. doi: 10.1371/journal.pone.0194240 (PMC5863987; doi:10.1371/journal.pone.0194240)
Supplement: S3 File — (PDF) [file pone.0194240.s003.pdf]

Interview guide

Participant #------------------------ Interviewer: -----------------------------

Date: -----------------------------

Part 1: Socio-demographic Characteristics

Age: ------------years--------------months Education: ---------------------------------

Gender: ------------------------------------- Income: ------------------------------PkR

Number of Family Members: --------------

Part 2: Informations about the users

Q 1: Who is drug user? (Please tick ✓ the relevant)

Your-self ☐ Family member ☐ Neighbor or relative ☐ Other ☐

Q 2: What is the gender of drug user? (Please tick ✓ the relevant)

Male ☐ Female ☐ Other ☐

Q3: In the case of female: have pregnancy or involved in breast feeding? (Please tick ✓ the relevant)

Pregnancy ☐ Breast feeding ☐ Nothing ☐ Other ☐

Q 4: What is the age of drug user? : ---------------------------years----------------------------months

Q5: Drug user have good health (in the case of more than 60 years*) ? **Can do himself / herself daily activities like walk and other physical activities, easily?* ------Yes ☐ No ☐

Q 6: Either drug user has any other disease/diseases or using any other treatment? (Please mention) : ---
----

Part 3: Practicing information

Q 7: What is patient's or your perception about the symptom or disease (For which you or patient preferred self-medication) : ---
----

Q 8: What is the reason of self-medication? (Please tick ✓ the relevant)

Affordability		Lack of confidence at doctor	
Access to hospital (24/7)		Privacy	

Emergency use		To save time	
Insignificant illness		*Other (Please mention below)	

*If other; please mention: ---

Q 9: How you got the informations about medicines? (Please tick ✓ the relevant)

By Media advertisements		From relatives, neighbors or friends	
From previous experience		From internet	
*Other (Please mention below)			

*If other; please mention: ---
