# Supplementary material for: Pattern of medication selling and self-medication practices: A study from Punjab, Pakistan
Source: PLoS One. 2018 Mar 22;13(3):e0194240. doi: 10.1371/journal.pone.0194240 (PMC5863987; doi:10.1371/journal.pone.0194240)
Supplement: S4 File — (PDF) [file pone.0194240.s004.pdf]

Permission and Information Sheet/ Patients

Sale of non-prescribed medicines and self-medication practices in Punjab, Pakistan

Interviewer: ______________________

Purpose of the study: The purpose of this study is to grasp the knowledge about the types of medicines purchased for self cure.

We will analyze:

What are socio-demographics characteristics of participants and drug users?

Why the people have self-medication?

What are the sources of information for self-medication?

It means to have in-depth understandings the behaviour of non-prescribed drug users.

.

Methodology: Observational study will perform to gather information about the attitude of people. We will conduct semi-structured interview to know societal behavior and reasons of self medication on your consent.

Confidentiality:

The interview will be questionnaire base. The information gathered from you will remain confidential and only the researchers will have access to it. Your name will not be used anywhere in the study. Data gathered from this study will be kept in a lock cabinet.

Before agreeing to this study, it is important that you have clearly understood the purpose of the study. This agreement states that you have understood everything about the study and that you are giving us permission to use information gathered from you for the study.

Date: _____________
