# Supplementary material for: Pattern of medication selling and self-medication practices: A study from Punjab, Pakistan
Source: PLoS One. 2018 Mar 22;13(3):e0194240. doi: 10.1371/journal.pone.0194240 (PMC5863987; doi:10.1371/journal.pone.0194240)
Supplement: S5 File — (PDF) [file pone.0194240.s005.pdf]

Permission and Information Sheet/ Pharmacy
Sale of non-prescribed medicines and self-medication practices in Punjab, Pakistan

Observer: ______________________

Purpose of the study: The purpose of this study is to grasp the knowledge about the types of medicines purchased for self-cure. To analyze the sale proportion of prescribed medicines and none-prescribed medicines in the province of Punjab. How the people ask for medicine for self-medication. It means to have in-depth understandings of drug sale pattern in community pharmacies.
.
Methodology: Observational study will perform to gather information about the sale of prescribed and non-prescribed medicines in your pharmacy during 2 full working days. We will also conduct a semi-structured interview of patients on their consents. .

Confidentiality:
The information gathered during this study will remain confidential and only the researchers will have access to it. Name of pharmacy will not be used anywhere in the study. Data gathered from this study will be kept in a lock cabinet.
Before agreeing to this study, it is important that you have clearly understood the purpose of the study. This agreement states that you have understood everything about the study and that you are giving us permission to use information gathered during this observational study.

Date: _____________
