# Supplementary material for: Pattern of medication selling and self-medication practices: A study from Punjab, Pakistan
Source: PLoS One. 2018 Mar 22;13(3):e0194240. doi: 10.1371/journal.pone.0194240 (PMC5863987; doi:10.1371/journal.pone.0194240)
Supplement: S6 File — (PDF) [file pone.0194240.s006.pdf]

Composition of Joshanada

Joshanda is a herbal product that contains:

Camellia sinensis,

Ephedra sinica,

Eucalyptus globules,

Foeniculum vulgare,

Glycyrrhiza glabra,

Hyssopus officinalis,

Judticia adhatoda and

Mentha piperita

It is manufacture by herbal companies on commercial basis and widely used herbal product in the community of Pakistan.
